# How do subvocal rehearsal and general attentional resources contribute to verbal short-term memory span?

**DOI:** 10.3389/fpsyg.2015.00145

**Published:** 2015-03-06

**Authors:** Sergio Morra

**Affiliations:** Department of Education, Università di GenovaGenova, Italy

**Keywords:** working memory, verbal short-term memory, attentional capacity, rehearsal, neo-Piagetian models, M capacity, order errors, short-term memory models

## Abstract

Whether rehearsal has a causal role in verbal STM has been controversial in the literature. Recent theories of working memory emphasize a role of attentional resources, but leave unclear how they contribute to verbal STM. Two experiments (with 49 and 102 adult participants, respectively) followed up previous studies with children, aiming to clarify the contributions of attentional capacity and rehearsal to verbal STM. Word length and presentation modality were manipulated. Experiment 1 focused on order errors, Experiment 2 on predicting individual differences in span from attentional capacity and articulation rate. Structural equation modeling showed clearly a major role of attentional capacity as a predictor of verbal STM span; but was inconclusive on whether rehearsal efficiency is an additional cause or a consequence of verbal STM. The effects of word length and modality on STM were replicated; a significant interaction was also found, showing a larger modality effect for long than short words, which replicates a previous finding on children. Item errors occurred more often with long words and correlated negatively with articulation rate. This set of findings seems to point to a role of rehearsal in maintaining item information. The probability of order errors per position increased linearly with list length. A revised version of a neo-Piagetian model was fit to the data of Experiment 2. That model was based on two parameters: attentional capacity (independently measured) and a free parameter representing loss of partly-activated information. The model could partly account for the results, but underestimated STM performance of the participants with smaller attentional capacity. It is concluded that modeling of verbal STM should consider individual and developmental differences in attentional capacity, rehearsal rate, and (perhaps) order representation.

## Introduction

The role of phonological coding in verbal short-term memory (STM) has long been recognized (e.g., Conrad, 1964), but phonological coding does not necessarily imply rehearsal. The role of rehearsal, both for adults and children, is more controversial. According to some models, verbal STM span depends strictly on a time-limited articulatory rehearsal process (Baddeley et al., 1975). Other models (e.g., Schneider and Detweiler, 1987) also recognize a role of rehearsal, but do not consider it the capacity-limiting bottleneck. Still others deny that rehearsal has any causal role in verbal STM (Brown and Hulme, 1995). Individual differences could complicate the problem; Logie et al. (1996) observed a word-length effect (that they consider a marker of articulatory processing) in the majority of a large sample, but about one quarter of that sample remembered short and long words equally well. Developmental differences are also relevant. There is general agreement that children start to use subvocal rehearsal around the age of 7 (see Jarrold and Tam, 2011, for a review). This implies that younger children's STM span must rely on other resources and be limited by other factors, and only after that age could rehearsal efficiency contribute to performance on STM tasks.

Although early componential models emphasized domain-specific storage systems, more recent theories (Cantor and Engle, 1993; Cowan, 2001; Barrouillet et al., 2009) emphasize instead the role of general attentional resources in working memory (WM). These theories are often based on tasks (such as the “complex span” measures) that do not merely involve maintenance of information, but also some deeper processing. Nevertheless, it is clear that “simple” and “complex” span share sizable variance (Engle et al., 1999). In the context of these theories, one can pose the problem of the role of subvocal rehearsal in STM; for instance, is it an additional storage device that adds to the capacity of the attentional system, or is it just an epiphenomenon without a causal role in memory performance, or a strategic process that enhances optimal use of the attentional resources?

Neo-Piagetian theories also emphasize domain-general WM resources and the important effects of WM capacity growth on cognitive development (see Morra et al., 2008, for a review). Indeed, the neo-Piagetian approach to cognitive development anticipated the current attention-based theories of WM capacity. Pascual-Leone (1970) suggested that an average 3-year-old has sufficient attentional resources (M-capacity) to activate one Piagetian figurative or operative scheme (i.e., one chunk of declarative or procedural knowledge), and that capacity would increase on average by one unit every second year, until a capacity of 6 or 7 schemes is reached during adolescence.

In a neo-Piagetian theoretical framework, however, only few studies considered STM development and, in particular, the role of subvocal rehearsal. Case et al. (1982) suggested that both STM and WM capacity develop as a consequence of automatization and increasing speed of processing; however, in the light of new evidence, Case (1995) dismissed that hypothesis and favored a maturational view of capacity growth. Burtis (1982) tested successfully a model of memory for supra-span lists of chunkable and non-chunkable consonants, based on Pascual-Leone's theory. Morra (2000) revised Burtis's model to account for phonological processing and STM span of word lists. It was assumed that activation of the representation of each (unrelated) word demands one unit of M-capacity, and that subvocal rehearsal would ensure maintenance of order information; however, rehearsal itself, conceived as a procedure (an operational scheme), would require one unit of M-capacity to be activated. Consequently, children would use rehearsal only after having developed sufficient M-capacity to find convenient allocating part of it to the rehearsal scheme. Two experiments with fourth- and fifth-graders tested successfully the fit of that model to the data. In addition, individual-difference analyses showed that both M-capacity and articulation rate (a proxy for rehearsal speed) contributed to verbal STM. Subsequent studies on learning new words (Morra and Camba, 2009) and on specific language impairment (Im-Bolter et al., 2006) provided further support for the view that general-purpose attentional resources are involved in WM processing of phonological information.

In contrast with early models of “short-term storage,” which were very simple, in the last decades a number of formal models of STM were proposed (e.g., Anderson and Matessa, 1997; Page and Norris, 1998; Burgess and Hitch, 1999; Brown et al., 2000; Lewandowsky and Farrell, 2008). Usually, these models are quite detailed regarding at least some aspects of representation of the memory items, their activation, the dynamics of decay or interference, and the retrieval process. Modeling the intended aspects of the STM functions often involves several free parameters; for instance, Oberauer and Lewandowsky (2008) compare a subset of models that they consider “relatively simple, with no more than four free parameters” (p. 544). However, these models are often silent or generic regarding a role of attentional capacity limits, and they intend to account for the average performance in different experimental conditions, but disregard developmental and individual differences. Some of these models are very specific about the rehearsal process (e.g., Burgess and Hitch, 1999), perhaps at the cost of disregarding other short-term maintenance processes, but other models do not consider rehearsal at all. Both Burtis (1982) and Morra (2000) are somewhat outsiders with respect to the mainstream formal models. They both are mainly concerned with accounting for developmental and individual differences in terms of attentional capacity limits; in addition, Morra (2000) considers some aspects of phonological processing and rehearsal. These two models are also simpler (perhaps simplistic, but also very parsimonious), because they both include only one free parameter.

A goal of the current study is to refine the Morra (2000) model and, in this perspective, clarify further the relationship between attentional resources and subvocal rehearsal in verbal STM. That model is inherently developmental, because it is framed within a developmental, neo-Piagetian view of attentional resources (e.g., Pascual-Leone, 1987; Pascual-Leone and Johnson, 2011). The current study, however, is carried out with participants in that particular developmental stage that is young adulthood. This choice is due to three reasons. First, adults have a larger STM and are more likely than children to reach longer list lengths in a span procedure; therefore, this study has a potential to generalize the results of Morra (2000) to older participants and a range of higher scores. Second, with young adult participants, one can assume that rehearsal ability has reached its plateau, and therefore it is possible to study the relationship between attentional resources and rehearsal in their best condition, when both components are fully developed. Finally, a study with adults could facilitate comparison and dialog with recent theories of WM, which can draw on a large base of adult data.

### The Morra (2000) model: strengths and weaknesses

The model under consideration preserves the essential structure of a previous one by Burtis (1982), modified to account for the essential features of phonological processing and serial order of word lists. The basic ideas embodied in that model are relatively simple. It assumes that remembering short lists of words requires both “figurative schemes” and “operative schemes” (or, if one prefers to avoid Piagetian jargon, both declarative and procedural knowledge). The procedural knowledge involved in this task concerns three distinct operations: encoding, rehearsal, and retrieval. Each of them is represented in the model as a distinct operative scheme. The declarative knowledge involved in the task obviously concerns the words; each word is represented as a single figurative scheme, whose degree of activation causes the probability of recalling that word correctly. In addition, one figurative scheme is required to represent the end-of-list signal, whose meaning must be understood by a participant as indicating that the list is over and one can start recalling it.

Each participant is assumed to have limited attentional resources (M-capacity), sufficient to fully activate a given number of schemes. Processing takes place in distinct steps: one to encode each presented word, one to get the end-of-list signal, and one for each word retrieved. At each processing step, the model represents which operative and figurative schemes are fully activated with the limited attentional resources available to the participant. In case any relevant figurative schemes (representing presented words) exceed the participant's M-capacity, their activation starts decreasing. At each step, the activation of each figurative scheme “in excess” decreases by an amount proportional to the number of schemes that are losing activation; that is, because partly activated schemes could interfere with each other, the more schemes are partly activated, the more each of them will reduce its probability of being recalled correctly.

The role of the three operative schemes can also be described simply. In case the stimuli are presented visually, phonological encoding is an operation that requires controlled attention, and thus takes one unit of M-capacity; but with auditory presentation, encoding is automatic (e.g., Penney, 1989), i.e., does not demand any M-capacity. In this model, phonological encoding of auditory input is the only process that is assumed to be attentionally automatic. Rehearsal also requires some attentional effort (e.g., Guttentag, 1984), and thus is assumed to take one unit of M-capacity; it is assumed to serve mainly the function of keeping track of the order of those words, whose schemes are fully activated. Retrieval is also assumed to be effortful, and thus demands one unit of M-capacity. The assumption that each of these schemes, to be activated with attentional effort, demands the same amount of resources, i.e., one unit of M-capacity, is consistent with the general assumption (from Pascual-Leone, 1970) that every scheme is a functional totality and, as such, demands the same amount of attentional energy for complete activation.

The model includes two parameters. One represents the size of the participant's M-capacity, and it is not a free parameter, because M-capacity can be independently measured with specific tests (as Morra, 2000 did). The other parameter represent the decrease of activation of the schemes that exceed M-capacity; this is a free parameter (the only one in the model) that can be estimated from the data. For an example of how the model works, see Appendix A.

This model has a number of advantages. First, it is conceptually simple and parsimonious, because it includes only one free parameter, as explained above. Second, it was supported by the data, because it predicted fairly well the means and variances of children's performance in both modalities, with words of different lengths, both with span measures and supra-span lists. Moreover, the estimates of the only free parameter were quite consistent across experiments. Furthermore, the correlation between M-capacity and verbal STM resisted partialling out age and several cognitive abilities.

On the other hand, some limitations of the Morra (2000) study can be noted. First, the age range of the participants was rather narrow; it is still necessary to test whether that model is also valid beyond grade five. For this reason, it seems particularly useful to assess how the model generalizes to prediction of adults' performance.

The Morra (2000) model is also too simple under several respects. Of course, each model is a simplification of the modeled reality; but oversimplification is not a virtue, and some of its assumptions can be criticized from this point of view. For instance, the model assumes that all presented words are encoded correctly; it does not consider the possibility that occasional, though rare, lapses of attention cause a failure to encode a word. If all words were encoded correctly, then the model would imply that participants with an M-capacity of at least 5 schemes always recall correctly the lists of three words. However, occasional errors do occur even with very short lists. Therefore, the model should probably be improved by assuming that each word is encoded correctly with a certain probability, high but slightly smaller than 1.

More important, the Morra (2000) model assumes that rehearsal has the function of keeping track of word order; thus, if a participant uses rehearsal and all words are recalled correctly, then they are recalled in the correct order. This is clearly an oversimplification, because order errors do occur. The model should be modified, to allow for order errors occurring even in case of rehearsal.

Another possible weakness of that model is the use of a subtractive function to represent the decrease of activation over successive processing steps of some schemes that represent words. This could lead to paradoxical outcomes with long lists, such as some words reaching negative activation. Current models of sequential processing (e.g., Anderson and Matessa, 1997; Lewandowsky and Farrell, 2008) often use multiplicative or power functions to represent decrease of activation, and the Morra (2000) model can be modified accordingly.

Finally, one limitation of that study concerned the analysis of individual differences. Morra (2000) found that verbal STM correlated significantly with both M-capacity and articulation rate, but correlation does not imply causation. In particular, there is a debate on whether the correlation between articulation rate and verbal STM is indicative of a causal role of rehearsal in counteracting decay in STM (e.g., Lewandowsky and Oberauer, 2008). Their argument concerns the correlation between word length and STM span in the population's average performance, but it can easily be extended to interpretation of individual differences; both variables could be affected by a third one, or the causal relation could even be the reverse, with a good verbal STM enhancing speed of articulation. To clarify this issue, one could consider different models of linear structural relations, including opposite directions of the causal effect between articulation rate and verbal STM.

### The current study

This study extends to young adults the investigation, carried out by Morra (2000) in primary school children, on the roles of attentional capacity and subvocal rehearsal in STM. The main goal of this article is clarifying how attentional resources and rehearsal contribute to verbal STM. In the service of this main goal, two ancillary goals are also pursued. One is extending to adulthood the validity of the Morra (2000) model, as well as refining it; for instance, taking into account ordering errors. Therefore, analyzing errors is another ancillary goal.

A first experiment examined order and item errors in a STM span task, in relation to word length, presentation modality, and individual differences in articulation rate. A second experiment considered individual differences in attentional capacity and rehearsal rate, and their relationship to verbal STM span, also using structural equation modeling. Finally, a revised version of the Morra (2000) model was tested for fit to the data of Experiment 2.

## Experiment 1

This experiment explored the error types as a function of modality and word length, and the relation between error types and individual differences in articulation rate. In particular, its purpose was to examine the probability of order errors with lists of different lengths, in view of a subsequent revision of the model.

### Methods

#### Participants

The participants were 49 young adults who volunteered for this study (27 men, 22 women, mean age 21 years, age range 18–32 years), including 29 university students and 20 in the last year of a technical high school.

#### Materials

The stimuli were constructed from two sets of 12 words each, i.e., two-syllable and four-syllable words, matched for frequency and imagery value.

From each set of stimuli, we created the word lists for six memory span trials. Each span trial included a two-word list, a three-word list, etc., up to eight words, to be presented in order of increasing length. The words for each list were selected randomly, with the constraints that two consecutive lists (e.g., the 3-word and the 4-word list in the same trial) could not include the same word in the same position, that the final word in a list could not be the final word in the previous list, and that a pair of consecutive words in a list could not appear in the same consecutive order in the next list. All words appeared with approximately equal probability throughout the span trials.

In addition, we used (only for practice) a set of 12 three-syllable words, from which we constructed only two span trials.

For the speeded articulation task, we divided each set into four groups of three words.

#### Procedure of the speeded articulation task

The speeded articulation task was administered at the beginning of the session. All participants started it with the practice materials (3-syllable words), then half of them received the 2-syllable before the 4-syllable words, and the other half vice versa.

Each group of three words was presented in turn on the screen, and the participant was instructed to read them aloud five times in a row, as fast as possible. A voice-key triggered the computer's clock when the participant started uttering the first word, and the experimenter pressed a key to stop the time when the participant was uttering the last syllable of the fifth consecutive repetition. This procedure was used in previous research (e.g., Morra, 2000) and was reported to be highly reliable, also when compared with other methods (Nicolson and Fawcett, 1991).

#### Procedure of the short-term memory task

The words could be presented visually or acoustically. For each participant, three span trials for each word length were presented visually and three acoustically. Which trials were presented in each modality was counterbalanced over participants. Half participants started with auditory and half with visual presentation. Within each presentation modality, all participants first received a practice trial with 3-syllable words; then, half received the 2-syllable words before the 4-syllable, and half vice versa.

Presentation of each list was started by the participant pressing the spacebar; after a 500 ms pause, the words were presented at a rate of one per 1500 ms. In visual presentation, the words appeared in the center of a 15” monitor in black boldface 18 pt Courier-New font, with a white background; each word was presented for 1000 ms, followed by 500 ms blank screen. For auditory presentation, there were audio files of each word, pronounced by a male voice without any strong dialectal accent. A row of asterisks in visual presentation and a beep in acoustic presentation indicated the end of a list, after which the participant was required to recall the whole list orally. In case of correct recall, the following list (one word longer) was presented, otherwise the trial was discontinued and the participant moved to the next span trial.

#### Scoring

In the STM task, each list was scored as correct or incorrect. Span was measured in each trial as the number of words in the longest list correctly recalled. For each participant, the measures obtained in the three trials with the same word length and modality were averaged into a single span score.

In addition, each error was classified into one of the following categories:

– omission (e.g., list A-B-C-D was reported as A-B-D)
– substitution (e.g., list A-B-C-D was reported as A-B-E-D)
– intrusion (e.g., list A-B-C-D was reported as A-B-C-E-D)
– ordering (e.g., list A-B-C-D was reported as A-C-B-D, or A-C-D-B)

In case a participant made more than one error on a list, only the first error was considered, because the errors following the first one could be due to attempts to repair, or other processes not relevant to this paper. Thus, for instance, if the list A-B-C-D was reported as A-B-D-E, it was scored as an omission, because the word between B and D was missing and not replaced by another, incorrect one; instead, if reported as A-B-E-C, it was scored as an intrusion. Ordering errors, therefore, were defined as recalling a word before its actual position, and reporting the word that actually was in that position at a later point.

For most analyses it is convenient to simply distinguish ordering errors (when a participant reports correctly two or more words, but not in their correct order) from item errors (when one or more words are omitted, replaced, or added).

Because each trial was discontinued as soon as the participant made an error, there was a potential total of 588 errors to be analyzed, i.e., 2 modalities × 2 word lengths × 3 trials × 49 participants. (Actually, only 585 errors were analyzed, because a participant performed without error on 2 trials, and 1 error was unclassifiable.)

The articulation rate was measured in words/s.

### Results

The descriptive statistics are reported in Table 1. The ordering errors, which are the point of main interest of this experiment, were on average 2.08 per participant, i.e., 17.4% of all the errors.

**Table 1:** **Descriptive statistics for Experiment 1**.

	**Mean**	**s.d**.
**ARTICULATION RATE**		
2-syllable (words/s)	3.30	0.57
4-syllable (words/s)	2.15	0.32
**MEMORY SPAN**		
Visual 2-syllable	4.62	0.91
Visual 4-syllable	3.96	0.69
Auditory 2-syllable	4.70	0.72
Auditory 4-syllable	4.20	0.74
**ERRORS**		
Omissions	5.16	2.29
Substitutions	3.84	2.15
Intrusions	0.86	0.87
Ordering	2.08	1.68

N = 49 for all measures, except articulation rate, where N = 48, due to malfunctioning of the voice key with one participant.

A 2 × 2 repeated measures ANOVA of the span scores, with presentation modality and word length as factors, yielded significant results for word length, *F*_(1, 48)_ = 81.54; *p* < 0.001; η^2^ = 0.63, and modality, *F*_(1, 48)_ = 6.75; *p* < 0.02; η^2^ = 0.12; the interaction was nonsignificant, *F*_(1, 48)_ = 1.28. This replicated the well-known advantage for short words and auditory presentation.

The articulation rate for short and long words was highly correlated, *r*_(46)_ = 0.76; *p* < 0.001, which is further evidence of reliability. For correlational analyses these two variables were averaged into a single measure of articulation rate.

Also the span scores in the four conditions were correlated, ranging from *r*_(47)_ = 0.61 to *r*_(47)_ = 0.72, all *p*'s < 0.001, showing reliable individual differences in verbal STM span.

Articulation rate correlated positively with span measures: *r*_(46)_ = 0.40; *p* < 0.01 for long words presented visually, *r*_(46)_ = 0.36; *p* < 0.02 for long words presented acoustically, *r*_(46)_ = 0.37; *p* < 0.01 for short words presented acoustically, and for short words presented visually the correlation was marginally significant, *r*_(46)_ = 0.25; one-tailed *p* < 0.05. These positive correlations also replicate a classical finding.

Having replicated the word length effect and the correlation between articulation rate and span, one can test the strong prediction that a time-limited articulatory process accounts for span. The presumed capacity of the articulatory loop can be obtained as the ratio of span to articulation rate (Baddeley et al., 1975); this ratio was computed for each participant, and the means for long and short words were compared. In visual presentation the mean capacity of the loop would be 1.44 s for short words and 1.87 s for long words, *t*_(47)_ = 9.55; *p* < 0.001. In auditory presentation the mean capacity of the loop would be 1.46 s for short words and 1.99 s for long words, *t*_(47)_ = 11.79; *p* < 0.001. Because different estimates of the articulatory loop duration were obtained with different word lengths, the strong hypothesis of a time-limited articulatory loop fully accounting for verbal STM span can be discarded. This result agrees with other recent studies.

A repeated-measures ANOVA, with presentation modality and word length as factors, was carried out on ordering errors. A significant effect was found for word length, *F*_(1, 48)_ = 6.14; *p* < 0.02; η^2^ = 0.11. On average, participants made 1.24 ordering errors with 2-syllable words and 0.84 with 4-syllable words. The same analysis, carried out on the total of item errors (substitutions + omissions + intrusions) obviously also yielded a significant result for word length, *F*_(1, 48)_ = 6.17; *p* < 0.02; η^2^ = 0.11. The means were 4.71 item errors for 2-syllable words and 5.14 for 4-syllable words.

**Figure 1:**
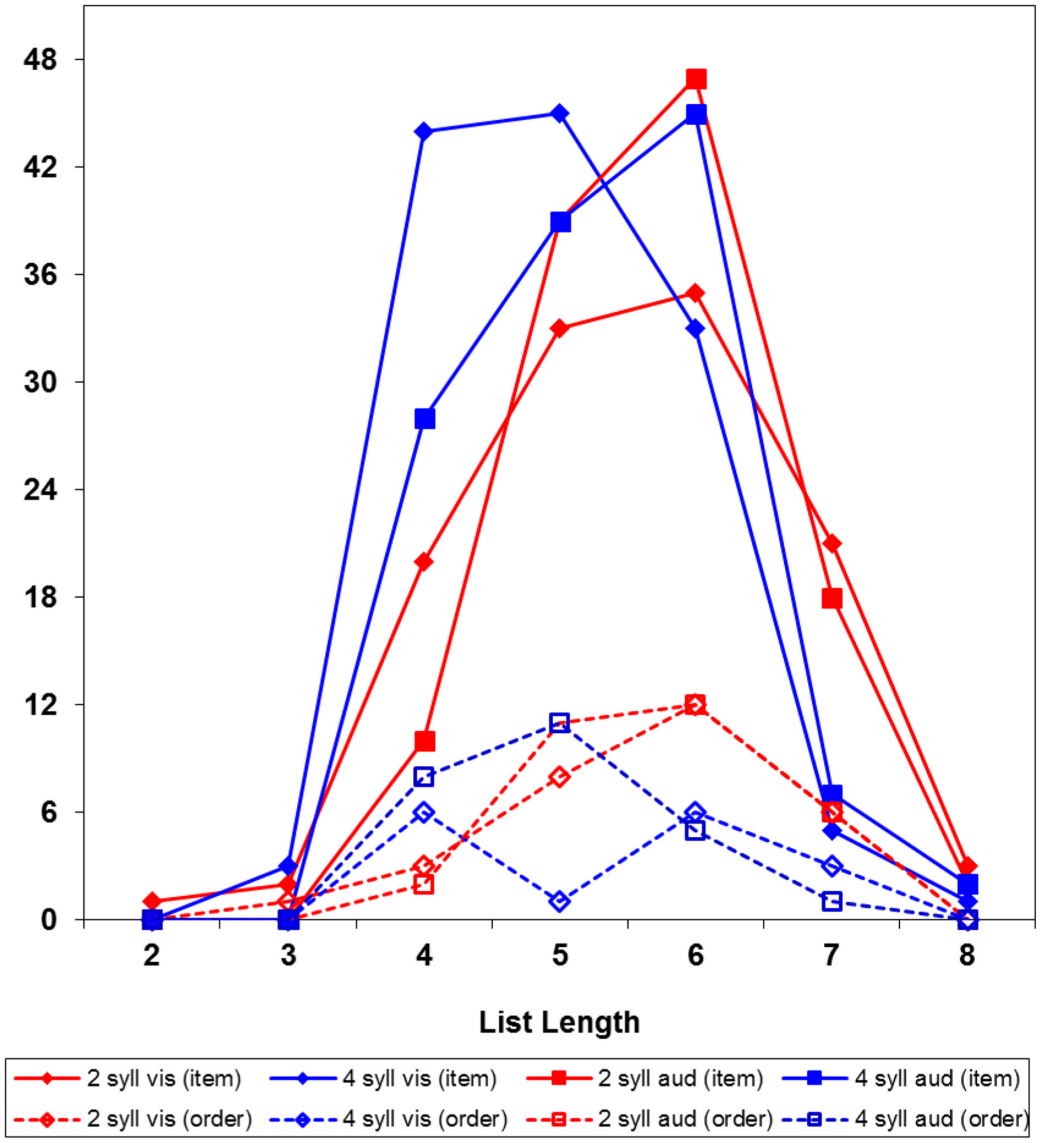
**Frequency of item and ordering errors by word length, modality, and list length**.

**Figure 2:**
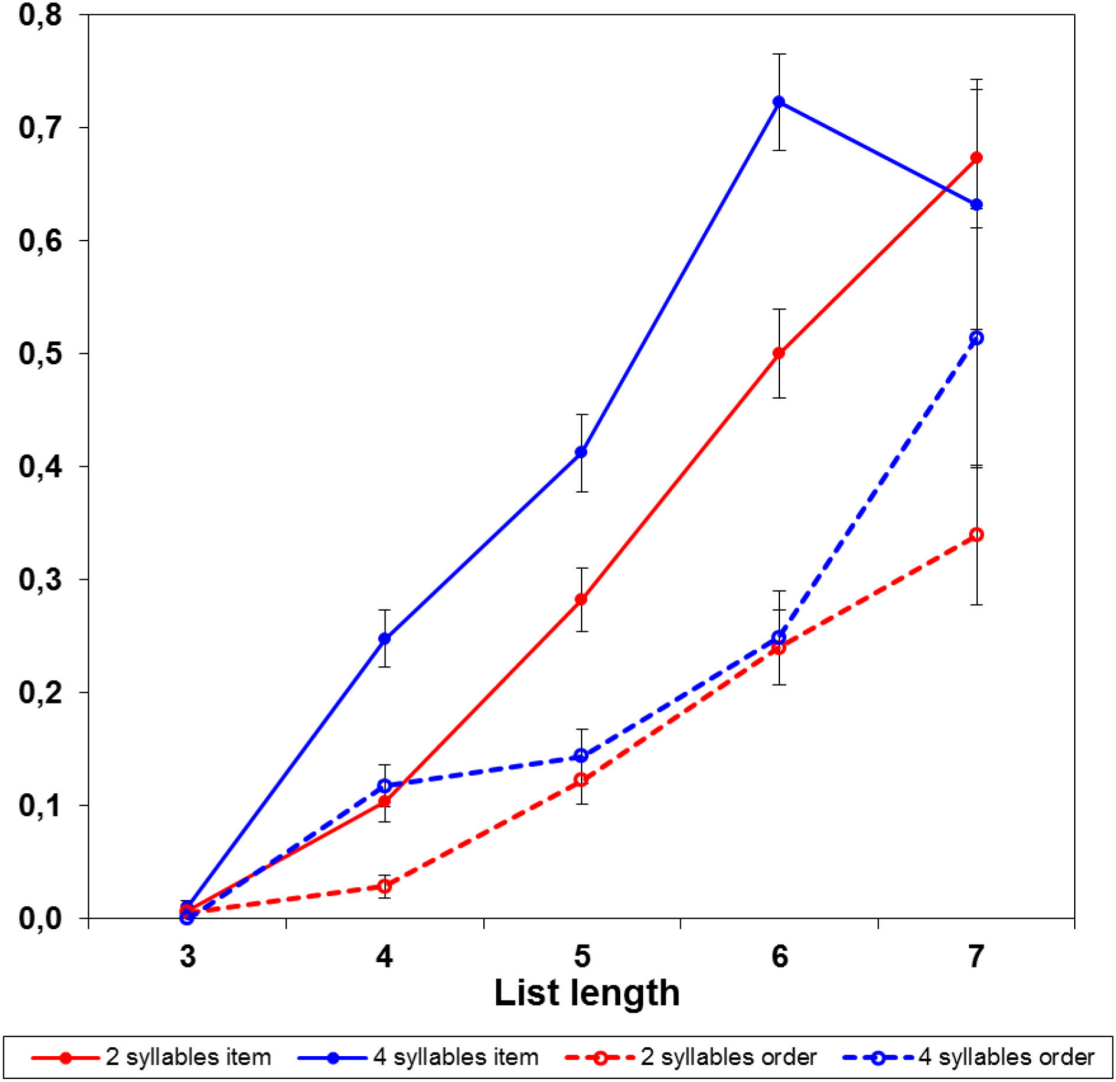
**Probability of item and ordering errors by word length and list length, controlling for the number of lists presented at each length**. Bars indicate the standard error of probabilities.

However, the fact that ordering errors were more frequent with short than long words does not imply that order of short words is harder to remember. The actual explanation is that the error on each trial was classified as either item or ordering error; with 4-syllable words there were more item errors, which prevented participants to proceed to longer lists, on which (as we shall see below) ordering errors are more probable. Figure 1 presents the frequency of item and order errors at each list length; note that, on 4-word lists, item errors were highly frequent with 4-syllable words, but not with 2-syllable words. Figure 2 presents the probability of item and order errors at each list length, controlling for the number of lists presented at each length; the item errors were more probable with 4-syllable than 2-syllable words. There was no evidence, instead, of ordering errors being more probable with 2-syllable than 4-syllable words. Therefore, the prevalence of item errors with long words was a reliable finding, but the prevalence of ordering errors with short words was just an artifact, due to discontinuing trials after an error.

Correlations of errors with articulation rate were computed. A positive correlation was found for ordering errors, *r*_(46)_ = 0.32; *p* < 0.03, and a negative correlation for item errors, *r*_(46)_ = −0.35, *p* < 0.02. Thus, a faster articulation rate was associated with decreasing numbers of item errors, and increasing numbers of order errors. Also in this case, of course, the two results are nearly specular.

A detailed analysis of ordering errors as a function of list length was carried out. There were 102 ordering errors in all, of which 96 were inversions of two consecutive positions, 2 were exchanges between non-consecutive positions, and 4 involved more than two positions. Thus, almost all ordering errors were inversions between two consecutive words. With the span procedure used here, 2-word lists were presented in all 12 trials to all participants, but with increasing list length, as errors were made and trials discontinued, the number of lists that were presented decreased. Thus, to analyze the probability of ordering errors at each list length, it is necessary to take into account the numbers of lists actually presented. Table 2 reports those numbers and the number of errors at each list length from 3 to 7 words (list length 8 is not considered, because very few trials continued up to that length). The probability of ordering errors increased with list length, but this is obvious, because more positions in a list imply more possible combinations of positions recalled in a wrong order. Given that ordering errors were essentially inversions of consecutive positions, in a 3-word list there are two possible inversions (positions 1–2 or 2–3), and in general, the number of adjacent pairs in a list equals the number of words minus one. A fair comparison of ordering errors across list lengths therefore requires that the error probability be divided by the number of adjacent pairs, i.e., by list length minus one. This corrected value is reported in the rightmost column of Table 2.

**Table 2:** **Probability of ordering errors as a function of list length in Experiment 1**.

**List length presented**	**Number of lists errors**	**Ordering**	**Probablity**	**p/(LL − 1)**
3 words	587	1	0.002	0.001
4 words	580	19	0.033	0.011
5 words	458	31	0.068	0.017
6 words	268	35	0.131	0.026
7 words	76	16	0.211	0.035

Probability = Number of ordering errors/Number of lists presented.

p / (LL − 1) = Probability / (List length − 1).

As can be seen, ordering errors were almost absent in 3-word lists, and increased linearly with list length. The regression equation, with a null intercept,

p/(LL−1)=0.009 (LL−3)

(where p is the probability of ordering errors at a certain list length and LL is the list length) accounted for a remarkable *R*^*2*^ = 0.998 of variance, *F*_(1, 4)_ = 1607.4; *p* < 10^−5^. Because ordering errors different from inversion of adjacent pairs were negligible, and the dependent variable is already corrected for the number of adjacent pairs in a list, this neat linear increase is not an artifact.

Because an ANOVA reported above found a different (although possibly artifactual) probability of ordering errors for short and long words, this regression analysis was also run separately for each word length. The B coefficient obtained was 0.009 (95% *CI* = 0.008, 0.010) for short words, and 0.009 (95% *CI* = 0.006, 0.011) for long words, with *R*^*2*^ = 0.991 and *R*^*2*^ = 0.958, respectively. Thus, we can conclude that the probability of ordering errors in a list, with these materials, was 0.009 (LL–3).

### Discussion

This experiment served well its purpose of estimating the probability of ordering errors. For lists of two or three words this probability can be estimated as zero, consistent with an assumption made by Morra (2000), that the first and the last position are salient and, if three words are recalled correctly, also the one in the middle position is identified by default. Starting from lists of four words, the probability of ordering errors per adjacent pair increased linearly with word length, and the linear regression equation reported above accounted for 99.8% of its variance. In case of error, most often, the word in the following position was recalled, and the error was “repaired” on the following position by recalling the word that was actually missed. The linear increase with list length of inversions per adjacent pair suggests that, in the range from list length four to seven, an increasing number of positions impairs discrimination of positions in the list. Therefore, in modeling Experiment 2, it will be assumed that, in case a participant correctly remembers all the words in a list, it is possible that on each position a word that was in another position is recalled, with a probability of 0.009 (LL–3), except on the final position (because, if all previous words were recalled in their correct positions, which one occupies the last position is determined by default).

This finding is important in view of a model that is valid throughout the life span. Because young children are less likely to reach long lists in a span procedure, having obtained from adults an estimate of the probability of ordering errors as a function of list length, this result could also be used in designing and modeling experiments with children.

In addition to estimating order error probability as a function of list length, this experiment explored the relations of different error types with other variables. Two novel results were found. Regarding experimental variables, item errors were more frequent with long words, and order errors with short words. Regarding individual differences, item errors correlated negatively and order errors correlated positively with articulation rate.

These two findings were somewhat unexpected, but they are jointly interpretable in a coherent way. Longer words are more difficult to rehearse, and therefore participants make a large number of item errors with 5-word and even 4-word lists; they make fewer item errors with short words, so they can proceed to longer lists, where the probability of ordering errors is higher. In addition, fast rehearsers are more effective at keeping word representations activated, so they make fewer item errors and proceed to longer lists, where the probability of ordering errors is higher. Slow rehearsers, instead, are more likely to fail on short lists because of item errors, and therefore, have fewer opportunities to incur in ordering errors on longer lists. Note that this interpretation entails that rehearsal serves to activate word representations—not to keep track of serial order.

The finding of a word-length effect on error types could be explained, alternatively, in terms of a greater interference among long words, which causes more item errors and, consequently, fewer opportunities for ordering errors. However, the negative correlation between articulation rate and item errors cannot be explained simply by interference. At least, one should make the additional assumption that rehearsal serves to counteract interference among word representations.

Because these findings are novel, future studies should ascertain how well they replicate to procedures different from the standard memory span, which involves a stop rule on each trial. It also remains to be studied whether they generalize to other materials and other languages, and of course, their developmental trends.

## Experiment 2

This experiment, replicating and extending the design of Experiment 1, examined the issue of attentional capacity and rehearsal as possible limiting factors of verbal STM span. Two structural equation models were compared, in which articulation rate was respectively hypothesized to be a causal factor of STM, or a by-product of it. In addition, the results of this experiment were fit to a revised version of the Morra (2000) model. The finding of Experiment 1 regarding the relation between list length and probability of ordering errors was used to set a fixed parameter representing this probability in the revised model.

### Methods

#### Participants

There were 102 young adults (19 men, 83 women, mean age 23 years, age range 18–41 years), of which 73 psychology students, who either volunteered for this study or participated for additional course credit.

#### Memory span measures

The materials and procedure used to measure STM span for short and long words in both presentation modalities were identical to Experiment 1.

#### Articulation rate measures

Also, the materials and procedure used to measure articulation rate for short and long words were identical to Experiment 1.

#### Attentional capacity measures

The measures of attentional capacity used in this experiment were tests constructed within the framework of Pascual-Leone's theory. This was also the case of those used by Morra (2000); however, the M-capacity measures used in that study were appropriate for children, whereas in this Experiment 3 tests suitable for adults were used (see also Pascual-Leone and Johnson, 2011). They are listed below.

#### Compound Stimuli Visual Information (CSVI) Task

The CSVI presents figures with a variable number of relevant features and the participant is required to respond to each of them, by pressing different buttons on a specifically designed keyboard. In a preliminary phase, the participant is trained to respond to each of nine features by using stimuli with a single relevant feature, until a criterion of perfect performance is reached. During the test phase, each stimulus has 2–8 relevant features; 56 stimuli are presented in pseudo-random order, for 5 s each, and the participant is not informed of how many relevant features each stimulus has. A probabilistic model based on the Bose-Einstein distribution was proposed by Pascual-Leone (1970) to account for performance on this task. The parameter *k*, representing a person's capacity (i.e., the number of schemes that can be activated simultaneously), was estimated in the range of integers 3–9 for each participant, as the value in the Bose-Einstein model fitting best his/her distribution of correct responses.

#### Direction Following Test (DFT)

The DFT (Cunning, 2003; Pascual-Leone and Johnson, 2005, 2011) requires the participant, on each item, to place one or two tokens (colored shapes) on the spaces of a special board. Items are in the form, e.g., “Place a small green circle and a large red square on a small yellow space,” and vary in syntactical complexity and in the number of features used to describe a token or a space. There are nine item types, constructed according to a theory-guided task analysis, so that they place different demands on M-capacity to understand and perform the instructions. The test comprises 5 practice and 45 test items. Because the sentence grammar varies across languages, Morra et al. (2013) validated an Italian version of the DFT, and scoring rules to estimate participants' M-capacity from it. The Italian version was used in this experiment.

#### Figural Intersections Test (FIT)

The FIT (Pascual-Leone and Baillargeon, 1994; Pascual-Leone and Johnson, 2011) includes 2 practice and 36 test items. Each item presents, on the right, a set of separate shapes. On the left, the same shapes (possibly rotated or changed in size) are presented as overlapping; in some items, an irrelevant figure is added to the overlapping set. The participant is required to find the intersection area of all the shapes presented on the right. The number of overlapping figures varies from 2 to 9 and is called the level of an item. The score was the highest consecutive level at which a participant responded correctly on at least 75% of the items, plus one point for any additional level at which at least 75% of the items were correct.

#### General procedure

Each participant was tested in three individual sessions, lasting approximately 45–60 min each. The CSVI was administered in the first session. Articulation rate and memory span were tested in the second session. In the third, the DFT and the FIT were administered.

### Results

Descriptive statistics are reported in Table 3. A 2 × 2 repeated measures ANOVA of the span scores, with presentation modality and word length as factors, yielded significant results for word length, *F*_(1, 101)_ = 121.86; *p* < 0.001; η^2^ = 0.55, modality, *F*_(1, 101)_ = 11.16; *p* < 0.002; η^2^ = 0.10, and the interaction, *F*_(1, 101)_ = 4.87; *p* < 0.03; η^2^ = 0.05.

**Table 3:** **Descriptive statistics for Experiment 2**.

	**Mean**	**s.d**.
**ARTICULATION RATE**		
2-syllable (words/s)	3.51	0.45
4-syllable (words/s)	2.30	0.27
**M CAPACITY**		
CSVI	6.21	1.61
DFT	6.53	1.36
FIT	6.50	1.42
**MEMORY SPAN**		
Visual 2-syllable	4.67	0.82
Visual 4-syllable	4.06	0.67
Auditory 2-syllable	4.76	0.71
Auditory 4-syllable	4.33	0.61
**ERRORS**		
Omissions	4.30	2.04
Substitutions	3.20	1.80
Intrusions	0.64	0.79
Ordering	3.48	1.93

N = 102 for all measures.

The modality and word length effects were also found in Experiment 1, but the interaction was nonsignificant, possibly because of smaller sample size and larger error variance. However, the pattern was nearly the same in both cases; in this experiment, the modality effect consisted of an auditory advantage of 0.27 long words but only 0.09 short words, and in Experiment 1 there was an auditory advantage of 0.24 long words but only 0.08 short words. As an additional check, given the identical method for span measures in both experiments, an ANOVA carried out on the pooled data yielded *F*_(1, 150)_ = 5.93; *p* < 0.02; η^2^ = 0.04 for this interaction, thus confirming that it was reliable, although the effect size was not large.

The ratio of span to articulation rate was computed for each participant and the means for long and short words were compared. In visual presentation the presumed mean capacity of the loop would be 1.35 s for short words and 1.78 s for long words, *t*_(101)_ = 15.78; *p* < 0.001. In auditory presentation it would be 1.37 s for short words and 1.90 s for long words, *t*_(101)_ = 20.62; *p* < 0.001. Again, the strong hypothesis of a time-limited articulatory loop fully accounting for verbal STM span can be discarded.

Similar 2 × 2 ANOVAs were carried out on memory errors (there were in all 439 omissions, 326 substitutions, 65 intrusions, and 355 ordering errors; in addition, there were 4 error-free trials, 1 unclassifiable error, and 33 trials with missing information for error type). For item errors, the significant word length effect found in experiment 1 was replicated, *F*_(1, 101)_ = 9.82; *p* < 0.01; η^2^ = 0.09, and the means were 3.84 item errors for 2-syllable words and 4.30 for 4-syllable words. The modality effect was also significant, *F*_(1, 101)_ = 4.10; *p* < 0.05; η^2^ = 0.04; participants made 3.92 ordering errors with auditory presentation and 4.22 with visual presentation. The interaction, *F*_(1, 101)_ = 0.02, was nonsignificant. Symmetrical results were obviously found for ordering errors; the word length effect was significant, *F*_(1, 101)_ = 8.45; *p* < 0.01; η^2^ = 0.08. On average, participants made 1.96 ordering errors with 2-syllable words and 1.52 with 4-syllable words. A significant effect was also found for modality, *F*_(1, 101)_ = 6.06; *p* < 0.02; η^2^ = 0.06. On average, participants made 1.92 ordering errors with auditory presentation and 1.56 with visual presentation. The interaction was not significant, *F*_(1, 101)_ = 0.05.

A repeated-measures ANOVA was used to compare the three tests of attentional capacity (CSVI, DFT, and FIT), which yielded *F*_(2, 202)_ = 2.02; *p* > 0.13; η^2^ = 0.02. The detail that these means did not differ significantly from one another is relevant, because these measures are not defined on arbitrary scales, but are assumed to represent the actual number of schemes that a participant can activate simultaneously on those tasks.

Articulation rate correlated negatively with item errors, *r*_(100)_ = −0.20, *p* < 0.05, and positively with ordering errors, *r*_(100)_ = 0.25; *p* < 0.02, replicating the similar findings of Experiment 1.

Table 4 reports the correlations between all STM span, articulation rate, and attentional capacity measures. All span scores in the four conditions correlated with one another, ranging from *r*_(100)_ = 0.44 to *r*_(100)_ = 0.61, all *p*'s < 0.001. The articulation rates for short and long words were highly correlated, *r*_(100)_ = 0.78; *p* < 0.001. The attentional capacity measures showed somewhat lower correlations, because the three tasks differ considerably in their content and requirements; tests of general resources, such as executive functions or M-capacity, cannot be “pure” measures, and are thus bound to correlate only weakly with one another (e.g., Morra, 1994; Miyake et al., 2000). Their correlations ranged between *r*_(100)_ = 0.17; one-tailed *p* < 0.05, and *r*_(100)_ = 0.30; *p* < 0.01; these values are in the range usually found in both children and adults (e.g., Morra et al., 2001). It is perhaps surprising that the one correlation that was only marginally significant involved the two tests that are purely visuo-spatial in content (FIT and CSVI); however, the DFT correlated significantly with each of them and thus lay sufficiently solid foundations for measurement of M-capacity as a construct.

**Table 4:** **Correlations between STM, articulation rate, and attentional capacity measures in Experiment 2**.

	**STM 2-syll. vis**.	**STM 4-syll. vis**.	**STM 2-syll. aud**.	**STM 4-syll. aud**.	**Art. rate 2 syll**.	**Art. rate 4 syll**.	**CSVI**	**DFT**	**FIT**
STM 2-syll. vis.	1	0.60^*^	0.58	0.46^*^	0.32°	0.24°	0.40^*^	0.37^*^	0.05
STM 4-syll. vis.		1	0.49^*^	0.44^*^	0.39^*^	0.32°	0.34^*^	0.43^*^	0.07
STM 2-syll. aud.			1	0.61^*^	0.30°	0.26°	0.19	0.47^*^	0.10
STM 4-syll. aud.				1	0.38^*^	0.31°	0.15	0.38^*^	0.14
Art. rate 2-syll.					1	0.78^*^	0.18	0.21°	0.16
Art. rate 4-syll.						1	0.13	0.17	0.13
CSVI							1	0.30°	0.17
DFT								1	0.29°
FIT									1

N = 102 for all measures.

* p < 0.001;

° p < 0.05, two-tailed.

**Figure 3:**
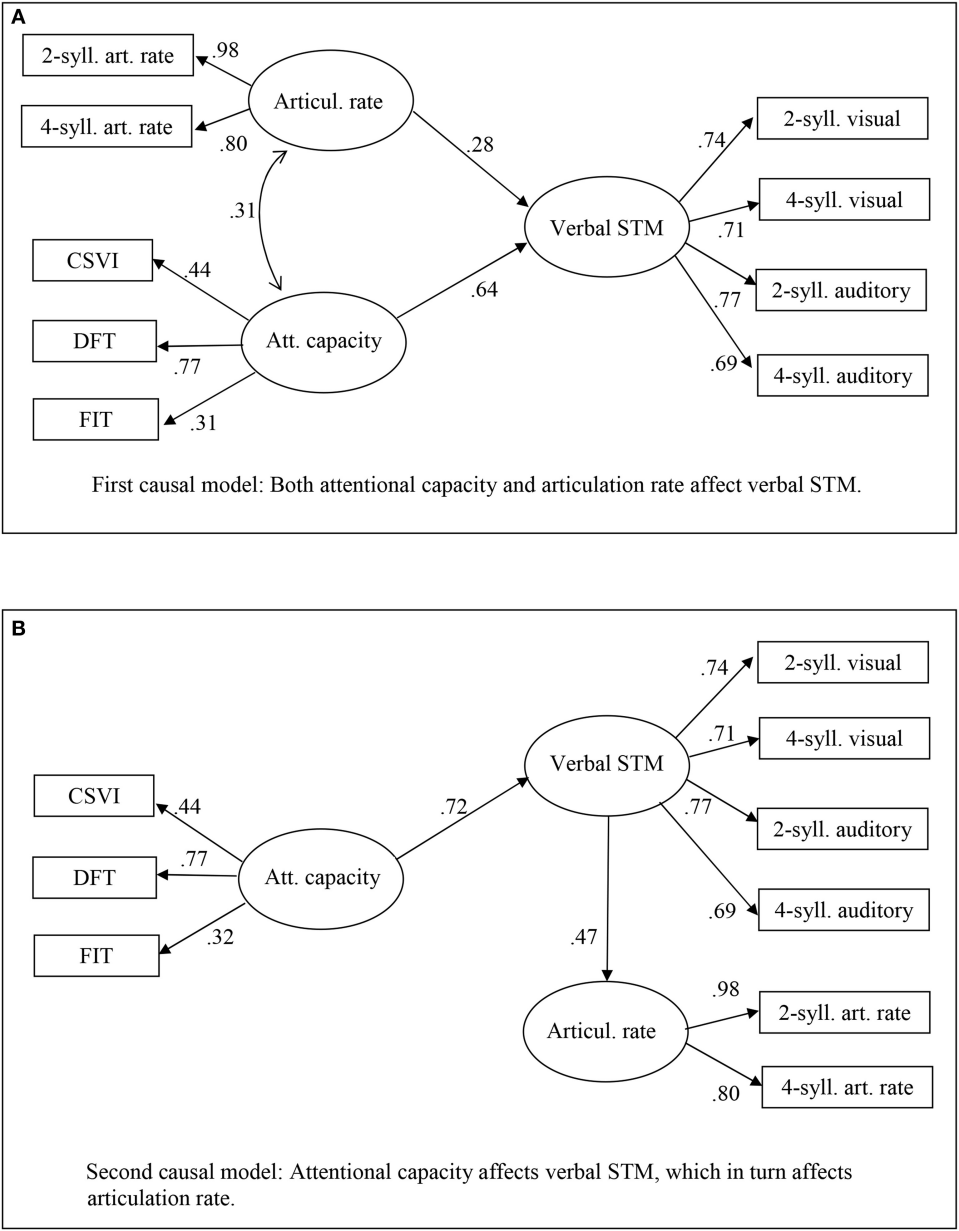
**Structural equation models for the variables measured in Experiment 2. (A)** First causal model: Both attentional capacity and articulation rate affect verbal STM. **(B)** Second causal model: Attentional capacity affects verbal STM, which in turn affects articulation rate.

This matrix of correlations was used for structural equation modeling. Preliminary analyses showed that the skew and kurtosis of each variable was adequate, and that a measurement model with three constructs (Verbal STM, Attentional capacity, and Articulation rate) fit the data well (*p* > 0.13). Hence, one can turn to test different models that posit specific causal relations among these constructs. Two such models are depicted in Figure 3.

The first model posits that both attentional capacity and articulation rate jointly affect verbal STM span (see Figure 3A). This model fits the data well, with a nonsignificant discrepancy between predicted and observed data, χ^2^_(24)_ = 31.76; *p* > 0.13, high goodness-of-fit indexes (*GFI* = 0.93; *AGFI* = 0.88; *CFI* = 0.99), and fairly low approximation errors and residuals (*RMSEA* = 0.057; *RMR* = 0.055). In this model, attentional capacity has a rather strong impact on verbal STM (*Gamma* = 0.64, *SE* = 0.15, *z* = 4.26, *p* < 0.001) and the articulation rate has a smaller, but significant impact on verbal STM (*Gamma* = 0.28, *SE* = 0.12, *z* = 2.40, *p* < 0.02).

The second model posits that attentional capacity affects verbal STM span, which in turn affects articulation rate (see Figure 3B). This model also fits the data well, with nonsignificant discrepancy between predicted and observed data, χ^2^_(25)_ = 31.80; *p* > 0.16, high goodness-of-fit indexes (*GFI* = 0.93; *AGFI* = 0.88; *CFI* = 0.99), and fairly low approximation errors and residuals (*RMSEA* = 0.052; *RMR* = 0.054). In this model, too, attentional capacity has a rather strong impact on verbal STM (*Gamma* = 0.72, *SE* = 0.15, *z* = 4.86, *p* < 0.001) and verbal STM, in turn, has a rather strong impact on the articulation rate (*Beta* = 0.47, *SE* = 0.11, *z* = 4.28, *p* < 0.001).

As one can note, either model seems to fit the data equally well. Because neither model is embedded in the other, it is not possible to test for significance of the χ^2^ difference. As an indirect comparison, one can note that some indexes are slightly better for the second than the first model; the differences, however, are indeed negligible.

### Discussion

The main goal of this experiment was assessing the roles of attentional capacity and rehearsal efficiency (represented, as a proxy, by articulation rate) as possible causes of individual differences in verbal STM span. Two different structural equation models were fit to the data for this purpose. The outcome was clear for attentional capacity, but ambiguous for rehearsal. Each model fit the data well, and in each of them attentional capacity emerged as a major determinant of verbal STM span, with regression coefficients of 0.64 in the first and 0.72 in the second model, respectively. This prominent role of attentional capacity is perhaps not so immediately evident from raw correlations, probably because each of the attentional tests has very different content and requirements; but using a latent variable, derived from extracting their common variance, permits the role of attentional capacity to emerge very clearly.

The fact that either model fit the data well, however, leaves it unclear whether articulation rate should be regarded as a cause or a consequence of individual differences in verbal STM span. If the first model is accepted, then we can consider rehearsal efficiency an additional, less massive but significant causal factor of STM. This pattern replicates Morra's (2000) findings in experiments with children—where structural equation models were not tested, but correlational analyses showed that M-capacity was a main predictor of verbal STM, and articulation rate was another predictor, accounting for a smaller but significant proportion of variance. However, an alternative view of the relation between verbal STM and articulation rate is equally acceptable, according to which a larger STM span would enable a faster articulation rate. Because the two models fit the data equally well, structural equation modeling is inconclusive on whether articulation rate should be viewed as a cause or a consequence of verbal STM span.

A conclusion, however, could be aided by cues provided by other results. One is the word length x modality interaction, indicating a larger modality effect for long words. This replicates a finding obtained by Morra (2000) with children. A plausible account of this finding is that availability of an acoustic stream is more useful for stimuli that are more difficult to rehearse. (Also in this case, however, an alternative account is possible; if one assumes that the word length effect is due to greater interference among longer words, then an auditory input would be advantageous for counteracting interference).

Furthermore, the error results obtained in Experiment 1 were replicated in this experiment; item errors occurred more frequently with long words, and articulation rate correlated negatively with item errors. As discussed with respect to Experiment 1, this correlation may point to a specific function of rehearsal, i.e., aiding maintenance of item information.

In sum, the results of this experiment point to a prominent role of attentional capacity, and are consistent with an additional role of rehearsal. Most of the results also lend themselves to other interpretations, alternative to a causal role of rehearsal; in particular, structural equation modeling was inconclusive as to whether rehearsal efficiency is a cause or a consequence of STM span. One result, i.e., the correlation of articulation rate with item errors, seems however to point more clearly to a causal role of rehearsal in maintaining item information.

## A revised model

Also drawing on the results of the experiments reported above, it is possible to formulate a revised version of Morra's (2000) model, and test its fit to the results of Experiment 2. Essentially, the following changes are made in the model:

- It is assumed that word encoding could fail, e.g., because of occasional lapses of attention. A parameter *c* will represent the probability of encoding each word correctly. In the following modeling *c* is fixed = 0.99.
- The possibility of order errors on each position, except the last, is represented with a parameter *r*, which (according to the results of Experiment 1) will be set at 0.009 times (list length−3).
- Because neither experiment found a negative correlation between articulation rate and ordering errors, it seems unlikely that rehearsal has the main function of keeping track of word order. This point will be considered further in the final discussion; for the moment, a generic operative scheme (of unspecified nature) for ordering is included in the model, instead of a more specific rehearsal operative scheme.
- A multiplicative function is used, instead of a subtractive function, to represent decreasing availability of the words that are losing activation. More specifically, it is assumed that the schemes activated with M-capacity are fully available, but the probability to retrieve the ones that exceed M-capacity is multiplied, at each step, by a free parameter *a* power the number of words that at that point are losing activation. The model does not specify to what extent decay or interference are involved in this decrease of activation and probability of retrieval; however, including in the function the number of partly activated schemes as an exponent of parameter *a* implies that interference is involved in this retrieval probability decrease.

One additional change is a consequence of a methodological detail of this experiment: because both in visual and in auditory presentation the end-of-list signal was conventional, we must assume that in neither modality encoding and understanding that signal is automatic.

Apart from these changes, the overall structure of the model already validated with children is retained. In particular, its two main features are unchanged: (a) Attentional capacity is allocated to both operative and figurative schemes, and its size is *not* represented as a free parameter, but it is independently measured with specific tests; (b) The model contains only one free parameter, called *a* in this article, which represents a coefficient that multiplies at each processing step the activation of a single, non-fully activated word. This coefficient is assumed to be smaller than 1, although it is reasonably expected to be close to 1.

The details of the revised model are presented in Appendix B. Table 5 summarizes the probability of recalling a list, for each list length, presentation modality, and participant's M-capacity (in the range from *e* + 5 to *e* + 7, which are typical values for young adults). Of course, the probability of reaching a certain span score is the product of the probabilities of recalling a list of a given length *and* all the shorter ones.

**Table 5:** **Probability of whole list correct recall, by participant's M-capacity, presentation modality, and list length**.

**Modality**	**List length**	**M-capacity**		
		***e* + 5**	***e* + 6**	***e* + 7**
Visual	3 words	*c*^3^ (1-0*r*)^2^ *a*^0^	*c*^3^ (1-0*r*)^2^ *a*^0^	c^3^ (1-0*r*)^2^ *a*^0^
Visual	4 words	*c*^4^ (1-1*r*)^3^ *a*^14^	c^4^ (1-1*r*)^3^ *a*^4^	c^4^ (1-1*r*)^3^ *a*^0^
Visual	5 words	*c*^5^ (1-2*r*)^4^ *a*^37^	c^5^ (1-2*r*)^4^ *a*^18^	c^5^ (1-2*r*)^4^ *a*^5^
Visual	6 words	*c*^6^ (1-3*r*)^5^ *a*^76^	c^6^ (1-3*r*)^5^ *a*^46^	c^6^ (1-3*r*)^5^ *a*^22^
Visual	7 words	*c*^7^ (1-4*r*)^6^ *a*^135^	c^7^ (1-4*r*)^6^ *a*^92^	c^7^ (1-4*r*)^6^ *a*^55^
Visual	8 words	*c*^8^ (1-5*r*)^7^ *a*^218^	c^8^ (1-5*r*)^7^ *a*^160^	c^8^ (1-5*r*)^7^ *a*^108^
Auditory	3 words	*c*^3^ (1-0*r*)^2^ *a*^0^	*c*^3^ (1-0*r*)^2^ *a*^0^	c^3^ (1-0*r*)^2^ *a*^0^
Auditory	4 words	*c*^4^ (1-1*r*)^3^ *a*^13^	c^4^ (1-1*r*)^3^ *a*^4^	c^4^ (1-1*r*)^3^ *a*^0^
Auditory	5 words	*c*^5^ (1-2*r*)^4^ *a*^33^	c^5^ (1-2*r*)^4^ *a*^17^	c^5^ (1-2*r*)^4^ *a*^5^
Auditory	6 words	*c*^6^ (1-3*r*)^5^ *a*^67^	c^6^ (1-3*r*)^5^ *a*^42^	c^6^ (1-3*r*)^5^ *a*^21^
Auditory	7 words	*c*^7^ (1-4*r*)^6^ *a*^119^	c^7^ (1-4*r*)^6^ *a*^83^	c^7^ (1-4*r*)^6^ *a*^51^
Auditory	8 words	*c*^8^ (1-5*r*)^7^ *a*^193^	c^8^ (1-5*r*)^7^ *a*^144^	c^8^ (1-5*r*)^7^ *a*^99^

### Testing for goodness of fit

The participants' M-capacity was estimated by averaging their scores in the CSVI, DFT, and FIT, and rounding the average to the nearest unit. In order to maximize the number of available data points and avoid small-sized groups, the participants with an estimated capacity of 4 units were included in the *e* + 5 group, and the participants with an estimated capacity of 8 units were included in the *e* + 7 group. There were 19, 35, and 48 subjects in the *e* + 5, *e* + 6, and *e* + 7 groups, respectively; each of them contributed 3 span scores in each of the four experimental conditions. Therefore, there were 306 data points in each condition, of which 57, 105, and 144 respectively from participants in each M-capacity group.

The best fitting values of parameter *a* were 0.9795 for visually presented short words, 0.9815 for acoustically presented short words, 0.9262 for visually presented long words, and 0.9588 for acoustically presented long words. Using these values, probability distributions of span scores were generated for each combination of word length, presentation modality, and M-capacity. Table 6 presents the expected and observed means, along with the relevant *t*-tests; Table 7 presents the expected and observed variances, along with the relevant Chi-squares.

**Table 6:** **Observed and expected STM span means in Experiment 2, by word length, presentation modality, and participants' M-capacity**.

**Modality**	**Word length**	**M-capacity**	**Obs. mean**	**Exp. mean**	***t***	**d.f**.	***p***
Visual	2 syllables	*e* + 5	4.32	3.98	2.64	55	<0.05
Visual	2 syllables	*e* + 6	4.38	4.49	−1.31	103	n.s.
Visual	2 syllables	*e* + 7	5.02	5.07	−0.55	142	n.s.
Visual	4 syllables	*e* + 5	3.56	3.30	2.04	55	<0.05
Visual	4 syllables	*e* + 6	3.91	3.79	1.55	103	n.s.
Visual	4 syllables	*e* + 7	4.31	4.51	−2.45	142	<0.05
Auditory	2 syllables	*e* + 5	4.44	4.11	3.20	55	<0.01
Auditory	2 syllables	*e* + 6	4.53	4.59	0.72	103	n.s.
Auditory	2 syllables	*e* + 7	5.06	5.14	−0.98	142	n.s.
Auditory	4 syllables	*e* + 5	4.12	3.62	4.40	55	<0.001
Auditory	4 syllables	*e* + 6	4.11	4.12	−0.02	103	n.s.
Auditory	4 syllables	*e* + 7	4.57	4.77	−2.37	142	<0.05

**Table 7:** **Observed and expected STM span variances in Experiment 2, by word length, presentation modality, and participants' M-capacity**.

**Modality**	**Word length**	**M-capacity**	**Obs. variance**	**Exp. variance**	**χ^2^**	**d.f**.	***p***
Visual	2 syllables	*e* + 5	0.92	0.84	62.13	55	n.s.
Visual	2 syllables	*e* + 6	0.77	1.10	73.74	103	<0.05
Visual	2 syllables	*e* + 7	1.31	1.38	137.16	142	n.s.
Visual	4 syllables	*e* + 5	0.95	0.30	179.53	55	<0.001
Visual	4 syllables	*e* + 6	0.67	0.54	130.85	103	n.s.
Visual	4 syllables	*e* + 7	0.91	0.72	180.76	142	<0.05
Auditory	2 syllables	*e* + 5	0.60	0.98	34.79	55	<0.05
Auditory	2 syllables	*e* + 6	0.67	1.22	57.44	103	<0.001
Auditory	2 syllables	*e* + 7	1.14	1.49	109.48	142	<0.05
Auditory	4 syllables	*e* + 5	0.74	0.55	76.58	55	n.s.
Auditory	4 syllables	*e* + 6	0.67	0.79	89.92	103	n.s.
Auditory	4 syllables	*e* + 7	0.99	1.00	142.94	142	n.s.

The p-values for chi-square tests are two-tailed, because the model could either underestimate or overestimate the variance of span scores.

As one can observe, the results were mixed. In six cases out of twelve, the model predicted well the distribution means (in four cases, with *t* < 1 and a difference of few hundredths between observed and expected means). However, in the other six cases there was a significant difference between observed and expected means (including one case with *p* < 0.001 and another with *p* < 0.01). The most important weakness of the model was its systematic underprediction of the STM of participants with an M-capacity of *e* + 5, with mean differences ranging between a quarter and half word. In addition, the model slightly overpredicted the performance of the *e* + 7 M-capacity group with long words. The model predicted well, instead, the performance of participants with M-capacity = *e* + 6, and (with short words) *e* + 7.

Regarding variance, as one can note in Table 7, in six cases the model predicted well distributions' variances, with nonsignificant chi-squares and, sometimes, an observed/expected variance ratio quite close to 1. In the other six cases, however, the chi-square test was significant, and in particular, there were two cases with *p* < 0.001. No systematic bias could be detected, however; i.e., considering the six significant chi-squares, in four cases the model underpredicted the distribution's variance and in two cases overpredicted it—in particular, the two cases with *p* < 0.001 were an overprediction and an underprediction.

In sum, this model was only partly successful in predicting the data; in particular, it made wrong predictions for the group with smaller M-capacity, which performed better than predicted by the model. On the one hand, this model is particularly parsimonious, with only one free parameter, and therefore, being able to predict the distribution means and variances in half cases can be viewed as a (half) success. On the other hand, this model is aimed at predicting not just a correlation between STM and M-capacity span, but also, more precisely, the actual size of STM span as a function of M-capacity; from this point of view, the underprediction of verbal STM means in the *e* + 5 group is an unsatisfactory outcome. The model considers individual differences in attentional capacity, but not the differences in rehearsal efficiency or ability to keep track of word order; these could perhaps be its faults, and this point will be taken up in the general discussion. It should also be noted that an earlier (and simpler) version of this model was more successful in accounting for children's performance (Morra, 2000), which could indicate that attentional capacity has a more massive explanatory role in children, whereas in adults other sources of differences may also have a relevant role that should be represented in the model.

## General discussion

The results of this study can be summarized in three points. First, structural equation models showed clearly a major role of attentional capacity (M-capacity) in determining individual differences in verbal STM span. They were inconclusive, instead, regarding the role of subvocal rehearsal, because two models in which articulation rate was either a cause or a consequence of verbal STM fit the data equally well. In both models, however, attentional capacity had a large effect on STM. This replicates the results obtained with primary school children by Morra (2000)—where structural equation modeling was not used, but the results showed a substantial correlation between verbal STM and M-capacity, and a smaller but significant correlation between verbal STM and articulation rate.

Second, a number of results obtained in both experiments are compatible with the idea that rehearsal plays a role (particularly in keeping item information activated), although not all of them are uniquely interpretable in this way. The interaction between word length and modality, such that the modality effect is larger for long words, replicates a similar finding with children (Morra, 2000) and suggests that availability of an acoustic stream is more useful for stimuli that are more difficult to rehearse. Order errors were more often associated to short words (and in Experiment 2, also to auditory presentation) and item errors were more often associated to long words (and in Experiment 2, also to visual presentation). The larger number of order errors in easier conditions is due to the fact that in these experimental conditions, with a span procedure^1^, participants reach longer lists, which afford ordering errors with a higher probability. But the association of item errors with longer words suggests that, with stimuli that render rehearsal difficult, item information is more likely to be lost. An alternative account of these findings is possible, in terms of interference; the modality × word length interaction could suggest that an acoustic stream is more useful to support redintegration of long words, which suffer from greater reciprocal interference, and the larger number of item errors with long words could be explained in the same way. However, one finding cannot be interpreted merely in terms of interference; correlational analyses showed that, in both experiments, the participants with a higher articulation rate commit fewer item errors. This finding can hardly be interpreted in terms of interference alone; at the very least it suggests that, if interference is a cause of loss of item information, then an efficient rehearsal is instrumental to counteracting it.

Third, the revised model proposed in this article was only partly successful in accounting for the data, and in particular, it underpredicted the performance of the participants' group with smaller attentional capacity. A comparison with Morra (2000), where a similar model accounted well for children's performance, may suggest that M-capacity, the key construct in the model, can account for the size of verbal STM better in children than in adults, who may also have additional sources of STM variability. Consequently, it is important to discuss which sources they could be.

A first possibility is that the specific contribution of rehearsal to STM span is larger in adults than in children. Assuming that the model shown in Figure 3A is correct, the specific contribution of articulation rate to individual differences in memory span (*Gamma* = 0.28) would be 8% variance accounted for, whereas the partial correlation reported by Morra (2000) between articulation rate and span, with M-capacity and age controlled for, was *r* = 0.17, i.e., only 3% variance accounted for. However, if we accept the view that rehearsal actually contributes to span, we should also specify how it does. Morra (2000) suggested that rehearsal might serve the role of maintaining order information without excessive attentional effort; however, the correlations of articulation rate with different error types, found in this study, seem to suggest instead that rehearsal aids item information maintenance. In addition, Farrell and Lelièvre (2009) clearly demonstrated that subvocal rehearsal is not involved in scanning sequentially the positions in a word list; therefore, it seems that it is not used to represent order information. It follows that, if rehearsal has any role, then it must concern item information. How do both rehearsal and attentional resources contribute to maintain item information? In the spirit of componential models, one could think of two distinct stores, such as a phonological loop and an episodic buffer (Baddeley, 2000); however, that solution could face a number of theoretical problems, such as, how would the cognitive system “know” when to address one or the other store to retrieve a word? How would the stores be coordinated?

Camos et al. (2009) also found, with different methods, that both attentional refreshing and rehearsal contribute to verbal STM, and pointed out that “though being independent, the two mechanisms can work jointly on the same memory traces” (p. 467). They suggested that memory representations are multimodal (i.e., not merely phonological, although phonological features are prominent), and that both attentional refreshing and subvocal rehearsal can contribute to their activation. These conclusions of Camos and colleagues, also supported by further studies (e.g., Mora and Camos, 2013), are in agreement with Engle et al's. (1999) tenets that WM is the activated part of long-term memory, and that subvocal rehearsal is not a structural component of WM, but rather, a strategy or an optional operation. A recent study on automatic activation of semantic information in STM (Campoy et al., 2014) also seems to support the view that memory representations are multimodal, and can be activated in various and synergic ways. In agreement with Camos et al. (2009), the results of this study can be understood as suggesting that both attentional resources and rehearsal jointly contribute to keep word representations activated, and both sources of activation contribute, though to a different extent, to individual differences in verbal STM span.

A possible implication for further modeling would be that, in addition to a parameter representing decrease of activation of the items that are not fully activated with attentional resources, another parameter (perhaps variable across age groups or individuals) would be needed, to represent rehearsal counteracting that activation decrease.

However, rehearsal rate is not the only source of individual differences that could be missing in the currently revised model, and necessary to account for developmental differences as well. A second possibility concerns order representation. Morra (2000) made the simplifying assumption that, if all words are remembered and the participant uses rehearsal, then also their order is correct. That simplification proved acceptable with children, but adults are more likely to reach longer lists in a span procedure, and (as Experiment 1 demonstrated) the probability of ordering errors increases at a very regular rate with list length. Consequently, a fixed parameter was set in the revised model to represent this probability. However, individual differences in the ability to represent order information were not considered, and it is quite possible that individuals (or age groups) differ in this respect. To model individual differences in order representation, however, we may need to understand better order representation itself.

Debates on this issue took new vigor from the Nineties (e.g., Henson et al., 1996; Marshuetz, 2005); early theories based on either item chaining or item-position associations tend now to be abandoned (see Lewandowsky and Farrell, 2008; Morra et al., 2009), while new views emerge, often based on more holistic representations of a sequence, such as a primacy gradient (Page and Norris, 1998), a spatial analog (Van Dijck et al., 2013; Guida, 2014), or a rhythmic pattern (Morra and Epidendio, submitted). It seems premature to make strong claims on which of these mechanisms could affect individual or developmental differences in the ability to keep track of order in a verbal STM task. In this study, the ordering function was only represented with a generic operative scheme and a fixed parameter for the probability of ordering errors. However, given the current state of the art, it seems likely that in the next future order representation will be the target of developmental, individual-difference, and experimental studies, which hopefully will also inform the construction of more refined formal models.

Despite limitations of the revised model presented in this article, a strong conclusion that can be drawn is the major importance of attentional resources (M-capacity) in determining individual differences in verbal STM span—a result that extends to adulthood the evidence reported in earlier developmental studies (Burtis, 1982; Morra, 2000). This is also consistent with growing evidence supporting the existence of domain-general, capacity-limited attentional resources that have a central role in a variety of WM tasks (e.g., Kane et al., 2004; Saults and Cowan, 2007; Vergauwe et al., 2012; Majerus et al., 2014). Moreover, there is increasing agreement that developmental growth of such central resources is essentially maturational (Case, 1995; Pascual-Leone and Johnson, 2011; Cowan et al., 2014). Hence, this study strengthens the view that central, attentional resources should not be disregarded in accounting for developmental and individual differences in verbal STM.

Future research can investigate more deeply the aspects of this study that do not afford unambiguous conclusions—essentially, the causal role of rehearsal. An experimental manipulation such as articulatory suppression, which impedes rehearsal, could be the appropriate technique. It is possible to make clear predictions on how articulatory suppression would alter the pattern of results obtained here in Experiment 2. If rehearsal has a causal role (as in the model depicted in Figure 3A), then articulatory suppression should render nonsignificant the *Gamma* parameter linking memory span to articulation rate. Instead, if individual differences in rehearsal speed are a by-product of STM size (as in the model depicted in Figure 3B), then there is no reason to expect that articulatory suppression affects the *Beta* parameter linking articulation rate to memory span. Furthermore, if the causal role of rehearsal consists in refreshing item information, then articulatory suppression should eliminate the negative correlation between item errors and articulation rate.

To summarize, this study extends to adults the conclusions drawn by Morra (2000) in a study with children, that both attentional resources and (to a lesser extent) rehearsal efficiency have a role in determining individuals' verbal STM span. However, this study also suggests that abilities different from attentional resources, such as rehearsal and (possibly) the processes involved in order representation, may have a larger role in adults than in children.

The revised model presented in this article is only partly satisfactory, being perhaps too simple (although parsimonious) with only one free parameter. Other models, such as those labeled as “mainstream” in the introduction, are certainly more detailed. However, they lack parameters or components that seem to be essential—most notably, a quantification of attentional capacity limits, and possibly, of individual and developmental differences in these limits. It is not claimed that the model presented here is conceptually better than the “mainstream” ones, or more satisfactory in terms of goodness of fit to the results. However, it can be suggested that future development of formal models of verbal STM should take into account such aspects as the massive contribution of limited attentional capacity to verbal STM, the developmental and individual differences in central attentional capacity, and perhaps the interactions of limited attentional capacity with rehearsal and order representation.

### Conflict of interest statement

The author declares that the research was conducted in the absence of any commercial or financial relationships that could be construed as a potential conflict of interest.

## Figures and Tables

**Table A1:**
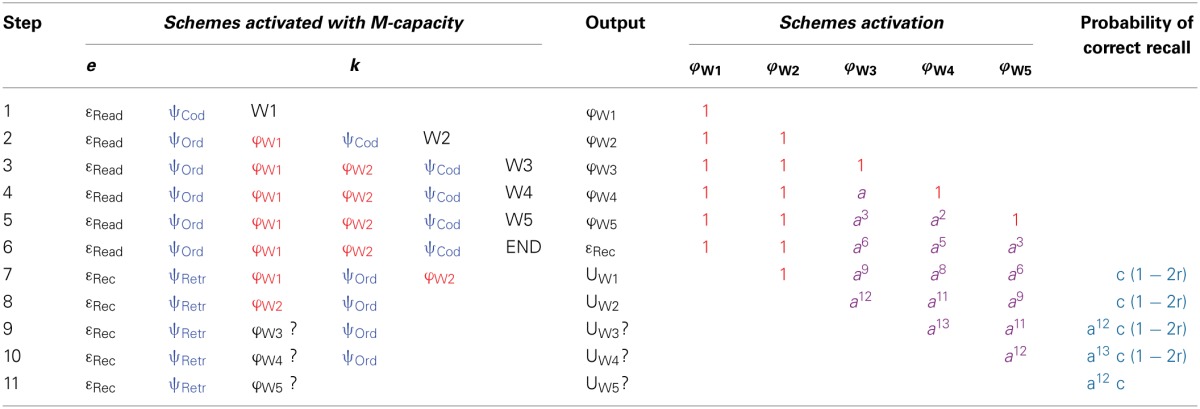
**Example of the revised model**.

Hypothetical sequence of processing steps by a participant with an M-capacity of e + 5, visually presented with a list of five words.

Probability of correct recall of the whole list = c^5^ (1 − 2r)^4^ a^37^.

Blue, fully activated operative schemes; red, fully activated figurative schemes; violet, degree of activation of fully activated figurative schemes; light blue, probability of recalling correctly each word.

## Footnotes

^1^If the procedure involves lists with a fixed number of words, long words yield both more item errors and more order errors than short words (see Mora and Camos, 2013). The point of interest in our results is that longer words tended to produce more item errors (rather than order errors). Follow-up experiments should assess how this result generalizes to different procedures and materials.

## Appendix A

### Example of the Morra (2000) model

This example is presented to clarify how the model works. Suppose that a participant who has the M-capacity necessary to activate five schemes (e.g., a typical 11-year-old) is presented visually with the list *ace, bike, corn, dog, elf*. On the first step, when the first word is shown, two units of M-capacity are used, one for the encoding operation and one to keep activated the phonological representation of *ace*. On the second step, the word *bike* is encoded while the word *ace* is rehearsed; M-capacity is allocated to four schemes (the encoding and rehearsal operatives, and the figurative schemes for the words *ace* and *bike*). Similarly, on the third step, five schemes are activated with M-capacity (the encoding and rehearsal operatives and the words *ace*, *bike*, and *corn*). At this point, the participant's M-capacity is exhausted (remember that we have assumed that the participant's capacity is limited to five schemes). On the next step, when the fourth word is presented, the participant can use one unit of M-capacity for the newly presented word *dog*, and the other four for the encoding and rehearsal operatives and the words *ace* and *bike.* The scheme for the word *corn* starts to lose activation, which drops from 1 (full activation) to 1 − *i* (where *i* is a free parameter). On the fifth step, the participant uses one unit of M-capacity for the newly presented word *elf*, and the other four for the encoding and rehearsal operative and the words *ace* and *bike.* The two words *corn* and *dog* lose activation, each of them by 2*i*, so that they drop to 1 − 3*i*, and 1 − 2*i*, respectively. Then (sixth step), the participant receives the end-of-list signal and spends one unit of M-capacity to understand it as a cue to start recalling the list, and uses the other four for the encoding and rehearsal operatives and the words *ace* and *bike.* The words *corn, dog*, and *elf* lose activation by 3*i*, and drop to 1 − 6*i*, 1 − 5*i*, and 1 − 3*i*, respectively. The seventh and eighth step involve recalling the words *ace* and *bike*, both of which are fully activated and thus can be safely retrieved. However, the three words *corn, dog*, and *elf* continue to lose activation, each of them by 3*i* both on the seventh and the eighth step, so that after retrieval of *bike* their activation has dropped to 1 − 12*i*, 1 − 11*i*, and 1 − 9*i*, respectively. On the ninth step, *corn* is retrieved with probability 1 − 12*i*, and meanwhile the schemes for the two words *dog* and *elf* keep losing activation, each of them by 2*i*, so that they drop to 1 − 13*i* and 1 − 11*i*, respectively. On the tenth step, *dog* is retrieved with probability 1 − 13*i*, and meanwhile activation of *elf* decreases by *i*, dropping to 1 − 12*i*. On the eleventh and final step, *elf* is retrieved with probability 1 − 12*i*. Therefore, the probability of recalling the entire list equals (1 − 12*i*) (1 − 13*i*) (1 − 12*i*).

In a similar way, given the participant's capacity limit, the list length, and the input modality, one can express the probability of recalling a list as a function of the free parameter *i*, which can be estimated from the data in each experimental condition (such as, for instance, auditory presentation of three-syllable words). It seems reasonable to assume that the parameter *i* will turn out to be larger for longer words, which are harder to rehearse, include more phonemes and thus can interfere more with each other, thus accounting for the well-known word-length effect.

## Appendix B

### Details of the revised model

Table A1 presents an example of how the revised model works. It refers to visual presentation of a list of five words, and its processing by a hypothetical participant with an M-capacity of *e* + 5 (i.e., the task executive plus 5 operative or figurative schemes). Because a number of symbols are used, it is convenient to explain their meaning first. W1, W2,… etc. stand for the first, second,… etc. word in a list. END stands for the end-of-list signal. The Greek letter ∈ stands for a relatively simple task executive (e.g., a representation of the current goal). As in Burtis (1982), ∈_Read_ and ∈_Rec_ represent the task executives for the input and recall phases of an STM task with visual presentation.

The letter ψ stands for an operative scheme. Three different operative schemes are included in the model. The first is ψ_Cod_, the word-encoding operation. The second is ψ_Ord_, an operation that keeps track of the order the currently activated figurative schemes that represent the words. The third is ψ_Retr_, the operation of retrieving the next word in a list.

The letter ϕ stands for a figurative scheme. In particular ϕ_W1_, ϕ_W2_, etc. stand for mental representations of the first, second,… etc. word in a list. The symbol ϕ_END_ stands for a mental representation of the end-of-list signal.

An important concept is the degree of activation of figurative schemes. On the right of Table A1, full activation of a scheme is indicated by the number 1, and decrease of activation is expressed in terms of *a*, the only free parameter in the model. This parameter is defined as the decrease in probability of recalling a word, per processing step and per decaying scheme. Therefore, decrease of activation depends both on the number of processing steps and on the number of partially activated schemes. Thus, if at a given point only one scheme exceeds M-capacity, then its activation is multiplied by *a* at that step; if two schemes exceed M-capacity, then the activation of each of them is multiplied by *a*^2^ at that step, and so on. The parameter *a* is assumed to be specific to each type of materials and experimental conditions (e.g., long or short words, digits, confusable or non-confusable consonants, semantic-conceptual codes; and visual or auditory input, fast or slow presentation, etc.).

Finally, U_W1_, U_W2_,… etc. represent utterances of recalled words. A question mark in the subscript, as in U_W5?_, indicates that the utterance of, for example, the fifth word occurs with a probability smaller than 1 because ϕ_W5_ is only partly activated.

During the first six steps, the subject has the goal of reading the stimuli (∈_Read_ is activated). In particular, at step 1, the subject has to encode (ψ_Cod_) the first word (W1). Only two of the subject's five units of M-capacity are used for this—one for the operation and one for the word. The outcome of this processing step is that a specific phonological code (ϕ_W1_) is activated at its highest degree (i.e., 1). At step 2, four M-capacity units are used. Two of them are allocated to the two schemes (ψ_Code_, W2) involved in phonological recoding of the second word, one keeps activated the scheme ϕ_W1_ of the previous word, and one (ψ_Ord_) is used to keep track of word order^2^. At this point, both ϕ_W1_ and ϕ_W2_ are fully activated. Step 3 is similar to step 2, except that five units of M capacity are used because two figurative schemes (i.e., ϕ_W1_ and ϕ_W2_) are now retained from the previous step; thus, ϕ_W1_, ϕ_W2_, and ϕ_W3_ are fully activated.

However, at step 4 it is not possible to continue activating one more scheme at its highest degree while keeping everything activated. To do so, an M capacity of *e* + 6 would be needed, but we are now modeling the processing of a subject with a capacity of *e* + 5, who can use two units of capacity for the operative schemes (ψ_Cod_, ψ_Ord_), two to keep activated the representations of the first two words (ϕ_W1_, ϕ_W2_) and one unit in recoding the current fourth word. The schemes ϕ_W1_, ϕ_W2_ and ϕ_W4_ are now fully activated, but activation of ϕ_W3_ begins to drop. Since only one scheme is decaying at this step, its activation is multiplied by the free parameter *a*, as shown in Table A1, step 4, in the ϕ_W3_ activation column.

Step 5 is similar to step 4, except that two articulatory codes, ϕ_W3_ and ϕ_W4_, are now losing activation. Therefore, the activation of each of them is multiplied by *a*^2^. Thus, activation of ϕ_W3_ drops from *a* to *a*^3^, and activation of ϕ_W4_ drops from 1 to *a*^2^.

On step 6 the conventional end-of-list signal is encoded, so that the participant sets him/herself the goal of recalling words. Activating the two relevant schemes (ψ_Cod_, END) uses two units of capacity. Therefore, two units can be used for the first two words (ϕ_W1_ and ϕ_W2_), and one for the word order (ψ_Ord_). Three schemes (ϕ_W3_, ϕ_W4_, and ϕ_W5_) are now losing activation, each by an amount of *a*^3^, dropping to *a*^6^, *a*^5^, and *a*^3^, respectively.

Steps 7–11 represent the recall phase. In particular, at steps 7 and 8 the first two words are safely recalled (with probability = 1), provided that they had been encoded correctly and that ordering errors do not occur, because they have been fully activated throughout the process. At step 7, M-capacity is allocated to the schemes ψ_Ord_, ψ_Retr_, ϕ_W1_, ϕ_W2_. On that step, activation of schemes ϕ_W3_, ϕ_W4_, and ϕ_W5_ continues to decrease by an amount of *a*^3^ because there are three partly activated schemes, and drops to *a*^9^, *a*^8^, and *a*^6^, respectively. Similarly, their activation still decreases at step 8, dropping to *a*^12^, *a*^11^, and *a*^9^, respectively.

At step 9, the third word is possibly recalled with probability = *a*^12^ (i.e., equal to the current degree of activation of ϕ_W3_). Meanwhile, ϕ_W4_ and ϕ_W5_ continue losing activation, so that at steps 10 and 11 the last two words are recalled with probability = *a*^13^ and *a*^12^, respectively.

The rightmost column of Table A1 summarizes the probability of recalling correctly each word, provided that each word had correctly been encoded (with probability *c*) and that recall of each consecutive word pair occurs in the correct order, which has probability 1 − (LL − 3) *r*, and under the assumption that at retrieval each word is recalled with probability equal to its degree of activation. In short, the probability of recalling correctly the whole list is the product of the probabilities of recalling serially each of its component words; in this example, it is *c*^5^ (1−2*r*)^4^ *a*^37^.

In the case of auditory presentation, it is assumed that phonological encoding is automatic (e.g., Penney, 1989) and, therefore, activation of ψ_Cod_ does not require attentional resources. Hence, one more unit of capacity is available to the participant throughout the input phase of the procedure.
